# Alterations in the Gut Microbiota and Metabolomics of Seafarers after a Six-Month Sea Voyage

**DOI:** 10.1128/spectrum.01899-22

**Published:** 2022-10-05

**Authors:** Chun-Hui Jiang, Xue Fang, Wen Huang, Ji-Yao Guo, Jia-Yun Chen, Hong-Yu Wu, Zhao-Shen Li, Wen-Bin Zou, Zhuan Liao

**Affiliations:** a Department of Gastroenterology, Digestive Endoscopy Center, Changhai Hospital, Shanghai, China; b Shanghai Institute of Pancreatic Diseases, Shanghai, China; Shandong University

**Keywords:** sea voyage, gut microbiota, 16S rRNA gene sequencing, untargeted metabolomics, seafarers’ health care, metabolic pathways

## Abstract

Maintaining the health of seafarers is a difficult task during long-term voyages. Little is known about the corresponding changes in the gut microbiome-host interaction. This study recruited 30 seafarers undertaking a 6-month voyage and analyzed their gut microbiota using 16S rRNA gene sequencing. Fecal untargeted metabolomics analysis was performed using liquid chromatography-mass spectrometry. Significant changes in the composition of the gut microbiota and an increased ratio of *Firmicutes*/*Bacteroidetes* at the end (day 180) of the 6-month voyage, relative to the start (day 0), were observed. At the genus level, the abundances of *Holdemanella* and *Plesiomonas* were significantly increased, while the abundance of *Bacteroides* was decreased. Predicted microbial functional analysis revealed significant decreases in folate biosynthesis and biotin metabolism. Furthermore, 20 differential metabolites within six differentially enriched human metabolic pathways (including arginine biosynthesis, lysine degradation, phenylalanine metabolism, sphingolipid metabolism, pentose and glucuronate interconversions, and glycine, serine, and threonine metabolism) were identified by comparing the fecal metabolites at day 0 and day 180. Spearman correlation analysis revealed close relationships between the 14 differential microbiota members and the six differential fecal metabolites that might affect specific human metabolic pathways. This study adopted a multi-omics approach and provides potential targets for maintaining the health of seafarers during long-term voyages. These findings are worthy of more in-depth exploration in future studies.

**IMPORTANCE** Maintaining the health of seafarers undertaking long-term voyages is a difficult task. Apart from the alterations in the gut microbiome and fecal metabolites after a long-term voyage, our study also revealed that 20 differential metabolites within six differentially enriched human metabolic pathways are worthy of attention. Moreover, we found close relationships between the 14 differential microbiota members and the six differential fecal metabolites that might impact specific human metabolic pathways. Accordingly, preventative measures, such as adjusting the gut microbiota by decreasing potential pathobionts or increasing potential probiotics as well as offsetting the decrease in B vitamins and beneficial metabolites (e.g., d-glucuronic acid and citrulline) via dietary adjustment or nutritional supplements, might improve the health of seafarers during long-term sea voyages. These findings provide valuable clues about gut microbiome-host interactions and propose potential targets for maintaining the health of seafarers engaged in long-term sea voyages.

## INTRODUCTION

Long-term voyages are challenging tasks. During these voyages, seafarers are confronted with a variety of stressors, including exogenous stressors (e.g., ship motion, different climate zones, chemical exposures, biohazards, and radiation) and endogenous stressors (e.g., circadian disruption, sleep deprivation, and anxiety) (1). These physiological and psychological stressors have insidious effects on the health of seafarers. For example, seasickness, a type of motion sickness of varying severity, is a common problem, occurring among 20 to 60% of seafarers during a voyage (2). Research suggests that the three most frequent causes of illnesses on long-term voyages are gastrointestinal disorders (21.0%), musculoskeletal diseases (15.4%), and cardiovascular diseases (15.1%) (3). Additionally, mental disorders (e.g., anxiety, stress, and depression) are increasingly affecting seafarers (4). Limited medical sources and supports onboard make it necessary to adopt preventative measures to ensure the health of seafarers.

Dysbiosis, or compositional changes in the intestinal microbiota caused by exogenous or endogenous factors, is associated with suboptimal health or even diseases. Gut microbes profoundly influence host physiology and pathology, including metabolism, immune system homeostasis, nervous system regulation, and pathogen colonization resistance (5–8). However, genomic analysis of gut microbiomes decodes only the microbial composition and potential rather than their actual activities. Furthermore, genomics cannot determine the interplay among the host, diet, and gut microbiota.

Metabolomics could bridge this gap by providing a complementary functional readout of the microbiome and valuable information about dynamic microbiome-host interactions (9). Gut microbiota-associated metabolites (e.g., short-chain fatty acids [SCFAs], secondary bile acids, trimethylamine or tryptophan metabolites) are associated with host metabolic homeostasis, immunoregulation, cardiovascular health, and cognitive behaviors (10–12). In addition, diet-derived metabolites play a pivotal role in diet-microbiota-host cross talk. For example, indigestible carbohydrates (e.g., cellulose in plants) are a nutritional source for members of the microbiota encoding carbohydrate-active enzymes (CAZymes) (e.g., *Bacteroidetes*, *Firmicutes*, and *Actinobacteria*); they can modulate the composition and function of the gut microbiota by promoting the growth of adept bacteria, and their short-chain fatty acid derivatives have positive effects on host health (13–15). Metabolomic analysis is a powerful approach for identifying biomarkers linked with aberrant biological functions and host disease-related metabolic pathways (e.g., imidazole propionate-p38γ/p62/mTORC1-diabetes) (16).

Limited studies have explored the impacts of long-term voyages on the intestinal microbiota, and the available studies have used mainly gene sequencing approaches. Zhang et al. (17) and Srivastava et al. (18) found that the intestinal microbiome of seafarers was altered and that multiprobiotics could maintain microbiome homeostasis during long sea voyages. Zheng et al. (19) found that a long-term voyage significantly changed the oral mucosa, skin microbiome composition, and microbial functions of seafarers. However, few studies have employed the metabolomics approach to analyze the impacts of sea voyages on seafarers. Moreover, the complex microbiome-host interactions during long-term voyages remain largely unknown. Therefore, the current study is the first to reveal the gut microbe-host interplay during a long-term voyage by performing a multi-omics analysis of genomics and metabolomics. The aim of this study was to identify risk factors and propose potential targets that might be beneficial for the health of seafarers engaged in long-term sea voyages.

## RESULTS

### Sequencing of the gut microbiota.

Thirty seafarers were enrolled in this study, and fresh fecal samples were collected from each participant at the start (day 0) and end (day 180) of a 6-month sea voyage. 16S rRNA gene sequencing was performed on the fecal samples. On average, 72,836 reads per fecal sample that passed the quality control (QC) filter were obtained using an Illumina MiSeq platform. Through further sequence processing, 64,261 reads on average were obtained for the final analysis. The average sequence length was 412 bp. The reads were clustered into operational taxonomic units (OTUs) at a 3% genetic difference; an average of 844 OTUs were obtained. The OTU flower plot revealed that there were 47 common OTUs among all samples (see Fig. S1a in the supplemental material). Specaccum curve analysis showed that the slope became flatter to the right (Fig. S1b), indicating that the majority of the species had been sampled.

### Analysis of the diversity of the gut microbiome of seafarers.

**FIG 1:**
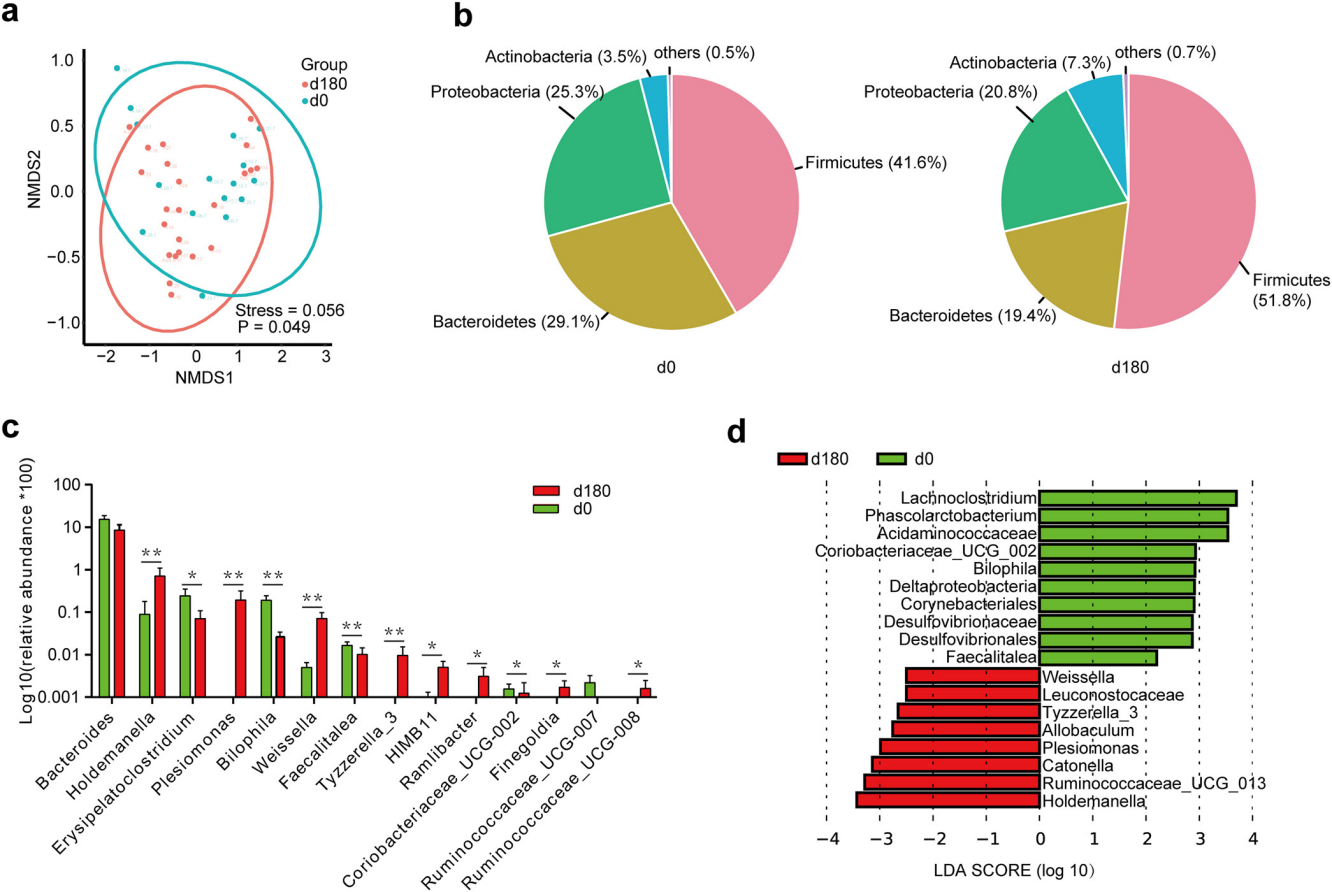
16S rRNA gene sequencing of bacteria in feces. (a) Nonmetric multidimensional scaling (NMDS) analysis based on the weighted UniFrac distance algorithm and the ANOSIM method between day 0 (d0) and day 180 of the voyage. The horizontal axis (NMDS1) and the vertical axis (NMDS2) are two sorting axes. (b) Pie charts of differences at the phylum level between day 0 and day 180 of the voyage. (c) Bar plot of the differential relative abundances of bacterial genera between day 0 and day 180 of the voyage. *, *P < *0.05; **, *P < *0.01. (d) Linear discriminant analysis effect size (LEfSe). Analyses of differential species abundances, evolutionary branches, and differential species scores between day 0 and day 180 of the voyage were performed. Red bars indicate relatively high-abundance species at the end (day 180) of the voyage, and green bars indicate relatively high-abundance species at the start (day 0) of the voyage.

Alpha diversity (Wilcoxon rank sum test) was analyzed based on the Chao1, Shannon, Simpson, and coverage indices as well as the phylogenetic diversity (PD) score. No significant changes were observed (Table 1). Rank-abundance curves also showed no significant differences (Fig. S1c). Nonmetric multidimensional scaling (NMDS) analysis based on the weighted UniFrac distance algorithm and the analysis of similarity (ANOSIM) method showed that there was a significant change in beta diversity (*P* = 0.049) after the 6-month voyage (Fig. 1a). This indicates that the long-term voyage altered the composition of the seafarers’ gut microbiota.

**TABLE 1:** Alpha diversity analysis by a Wilcoxon test between day 0 and day 180 of the voyage

Parameter	Value for day		*P* value
	0	180	
Chao1 index (interquartile range [25%, 75%])	924.641, 1,164.763	888.074, 1,058.560	0.39
Mean Shannon index ± SD	4.815646 ± 1.151	5.098 ± 0.852	0.30
Mean Simpson index ± SD	0.875 ± 0.082	0.908 ± 0.0515	0.18
Coverage index (interquartile range [25%, 75%])	595.675, 824.225	560.100, 903.400	0.14
PD score^*a*^ (interquartile range [25%, 75%])	26.593, 35.304	25.813, 39.775	0.94

a PD, phylogenetic diversity.

### Taxonomic analysis of the gut microbiome at the phylum and genus levels.

Phylogenetic analysis of the OTUs revealed that *Firmicutes* accounted for the majority of the microbial community, followed by *Bacteroidetes*, *Proteobacteria*, and *Actinobacteria* (Fig. 1b). The *Firmicutes*/*Bacteroidetes* ratio was increased after the voyage, but there was no significant difference (*P* = 0.151 [by a Wilcoxon rank sum test]). At the taxonomic level, log_10_ transformation of the relative abundance matrix was performed to show the differentially abundant bacteria between the start (day 0) and end (day 180) of the voyage. At the genus level, *Holdemanella*, *Plesiomonas*, *Weissella*, *Tyzzerella*_3, HIMB11, *Ramlibacter*, *Finegoldia*, and *Ruminococcaceae*_UCG-008 were significantly increased, while *Erysipelatoclostridium*, *Bilophila*, *Faecalitalea*, and *Coriobacteriaceae*_UCG-002 were significantly decreased after the voyage (Wilcoxon rank sum test) (Fig. 1c). The linear discriminant analysis (LDA) effect size (LEfSe) algorithm was applied to further evaluate the differences in abundances between the start (day 0) and end (day 180) of the voyage (Fig. 1d). As shown in the differential species score chart, *Holdemanella*, *Plesiomonas*, *Weissella*, and *Tyzzerella*_3 were among the relatively high-abundance species at the end (day 180) of the voyage, while *Coriobacteriaceae*_UCG-002, *Bilophila*, and *Faecalitalea* were among the relatively high-abundance species at the start (day 0) of the voyage.

### Predicted functional potentials of the changed microbiome.

**FIG 2:**
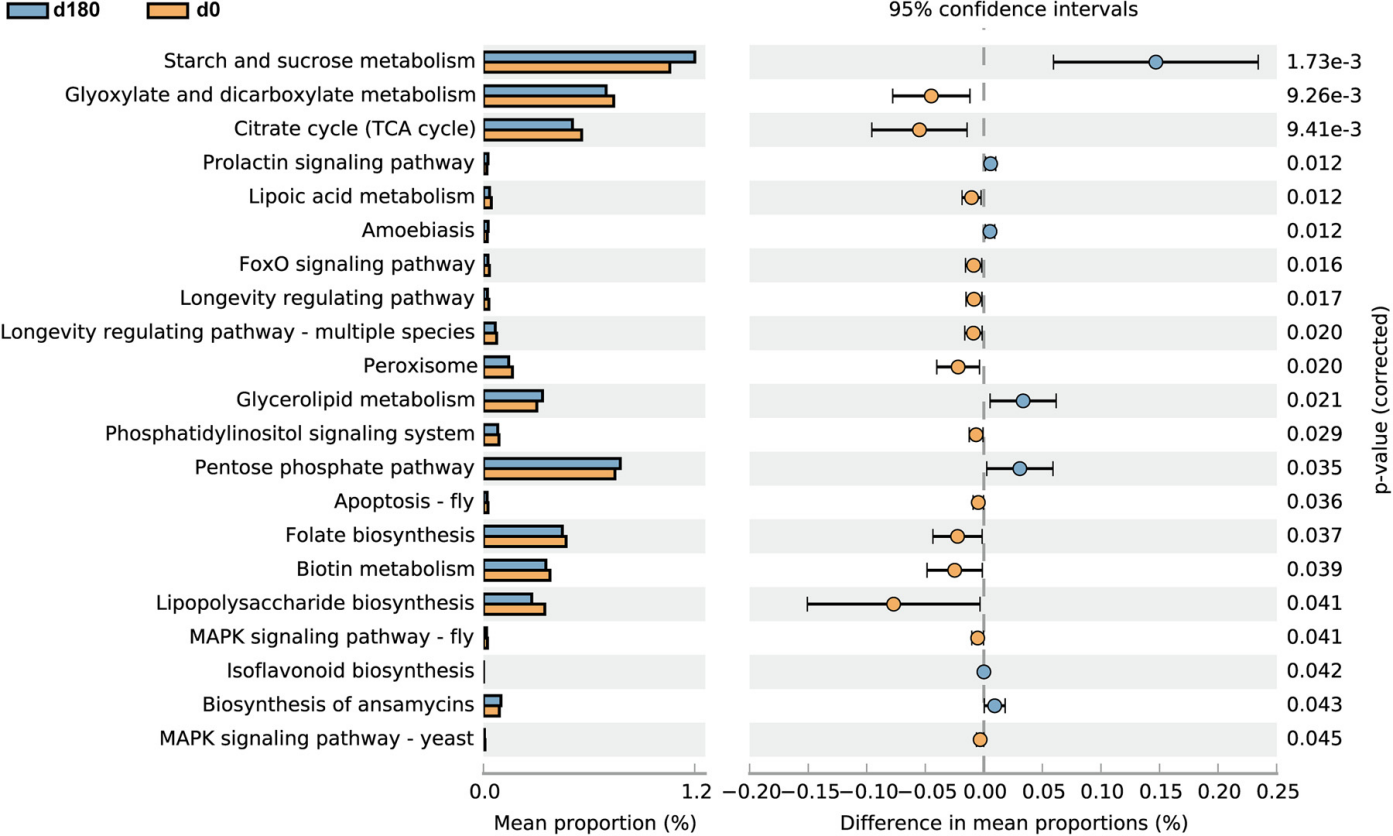
Predicted microbial functional analysis. Phylogenetic Investigation of Communities by Reconstruction of Unobserved States 2 (PICRUSt2) analysis resulting in Kyoto Encyclopedia of Genes and Genomes (KEGG) Orthology (KO) level 3 pathways between day 0 and day 180 of the voyage is depicted. MAPK, mitogen-activated protein kinase.

In order to predict the possible functions of changed genes in the gut microbiota, Phylogenetic Investigation of Communities by Reconstruction of Unobserved States 2 (PICRUSt2) was employed to determine Kyoto Encyclopedia of Genes and Genomes (KEGG) Orthology (KO) level 3 pathways. The results indicated that folate biosynthesis, biotin metabolism, the citrate cycle (tricarboxylic acid [TCA] cycle), and glyoxylate and dicarboxylate metabolism were significantly decreased while starch and sucrose metabolism, glycerolipid metabolism, and the pentose phosphate pathway were significantly increased after the voyage (Fig. 2).

### Liquid chromatography-mass spectrometry analysis of fecal metabolic profiles.

**FIG 3:**
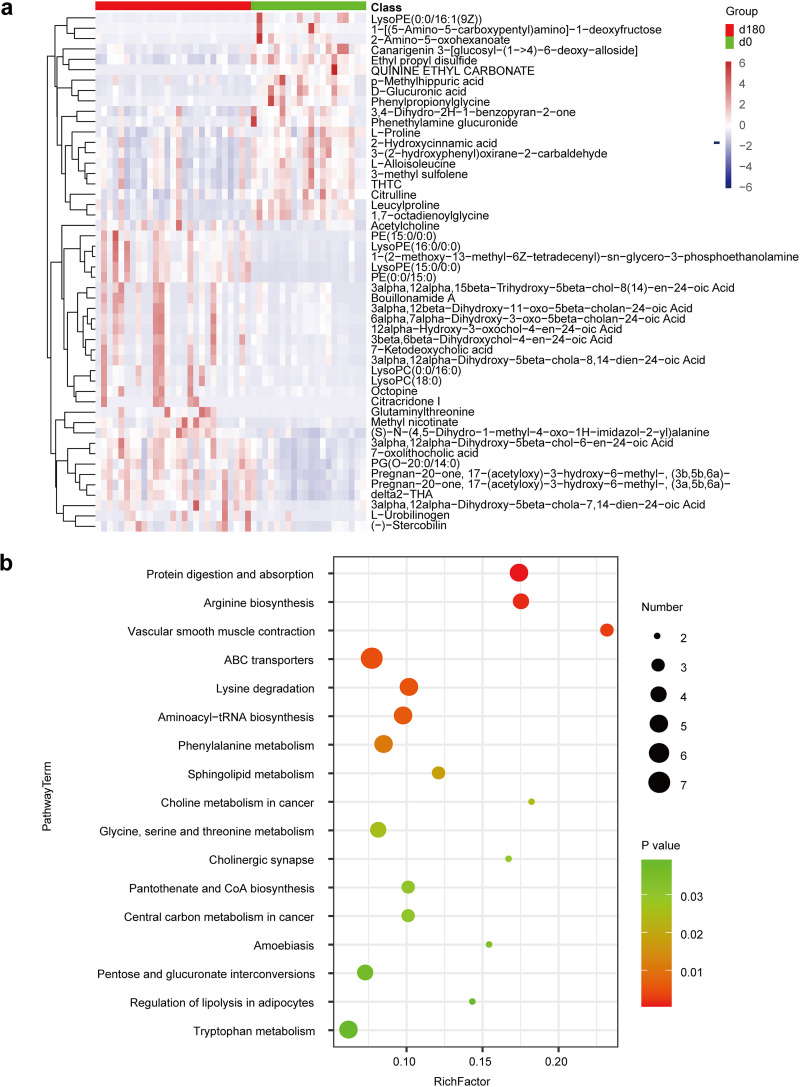
Liquid chromatography-mass spectrometry analysis of fecal metabolites. (a) Heat map showing the abundances of the top 50 differential metabolites that were significantly altered between day 0 and day 180 of the voyage. (b) Bubble chart of the top 20 enriched KEGG metabolic pathways between day 0 and day 180 of the voyage. The vertical axis is the metabolic pathway; the horizontal axis is the Rich factor (the Rich factor is the number of significant differential metabolites/the total number of metabolites in the pathway). The larger the Rich factor, the greater the degree of enrichment. Coloring from green to red indicates a decrease in the *P* value; the larger the point, the more metabolites enriched in the pathway; LysoPEs, lysophosphatidylethanolamines; THTC, 1-tetrahydrothiophenecarboxylic acid; LysoPC, lysophosphatidylcholine; PG, phosphatidylglycerol; THA, tetracosahexaeoic acid.

In order to determine the impacts of the long-term voyage on the seafarers’ fecal metabolites, liquid chromatography-mass spectrometry (LC-MS) untargeted metabolomics analysis was performed. Orthogonal partial least-squares discrimination analysis (OPLS-DA) effectively discriminated between the start (day 0) and end (day 180) of the voyage (Fig. S2a). The discrete points of the OPLS-DA S plot revealed the presence of significant differential metabolites between the start (day 0) and end (day 180) of the voyage (Fig. S2b). In total, 880 fecal metabolites were analyzed, and 143 differential metabolites were identified, the top 50 of which are shown in Fig. 3a. The differential metabolites included significant decreases in d-glucuronic acid, l-proline, 2-hydroxycinnamic acid, and citrulline and significant increases in acetylcholine after the voyage.

### Predicted impacts of the differential metabolites on human metabolic pathways.

**FIG 4:**
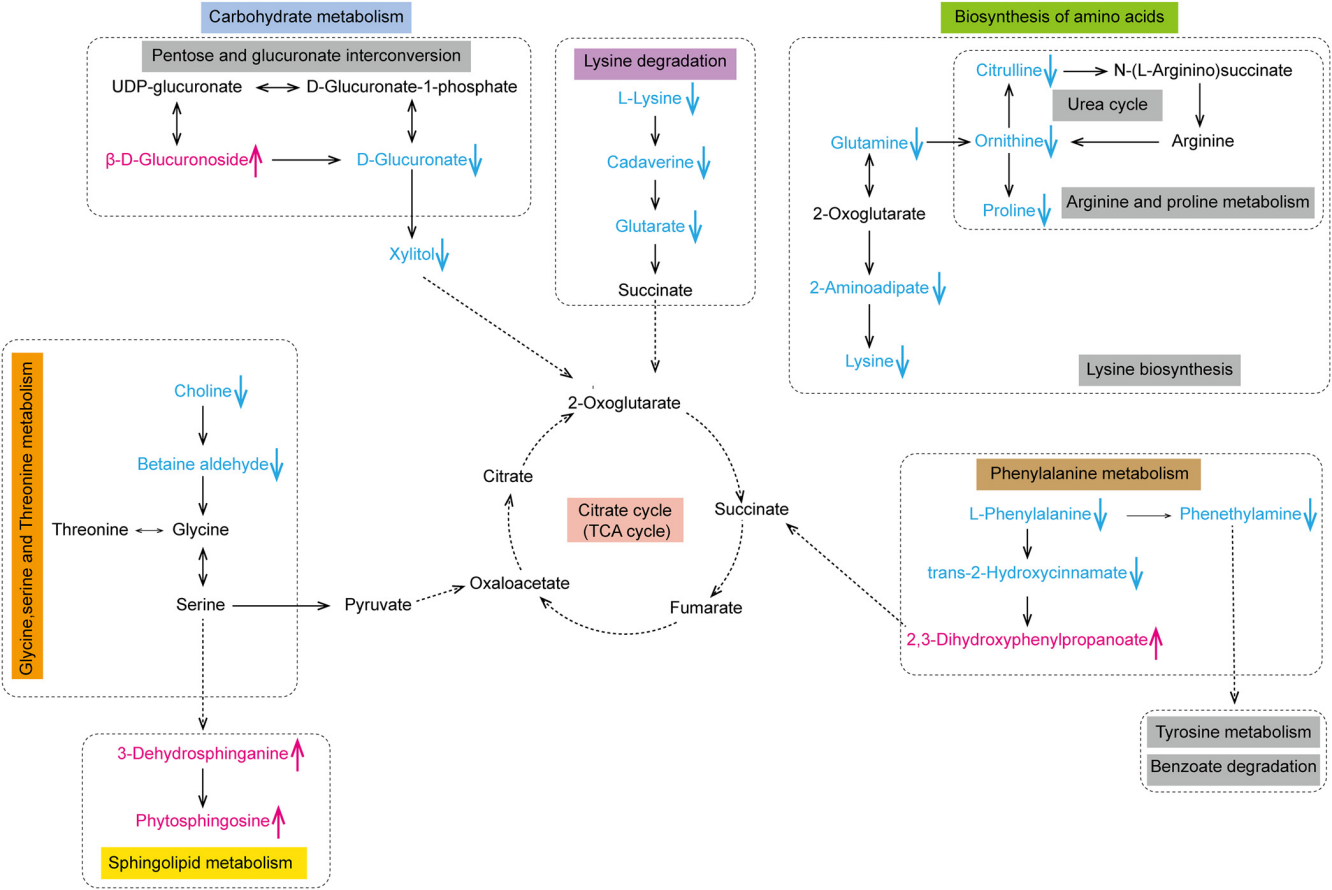
Schematic overview of the primary metabolic pathways affected between day 0 and day 180 of the voyage. Pink text indicates increased differential metabolites, and blue text indicates decreased differential metabolites.

The differential metabolites in the LC-MS analysis were then annotated according to the KEGG pathway mapper. A comparative analysis of the human metabolic profiles showed significant changes in protein digestion and absorption as well as changes in major metabolic pathways, including the carbohydrate, lipid, and amino acid pathways (Fig. 3b). Specifically, in carbohydrate metabolism, pentose and glucuronate interconversions were affected. In amino acid metabolism, arginine biosynthesis, lysine degradation, and glycine, serine, threonine, and phenylalanine metabolism were affected. In lipid metabolism, sphingolipid metabolism was affected. A schematic overview based on the reference map in the KEGG database was drawn (Fig. 4). We focused on 20 differential metabolites within six differentially enriched human metabolic pathways. Based on the *P* values, arginine biosynthesis, lysine degradation, phenylalanine metabolism, sphingolipid metabolism, pentose and glucuronate interconversions, and glycine, serine, and threonine metabolism were characterized as significantly relevant pathways affected by the long-term voyage; their *P* values were 0.0016, 0.0051, 0.011, 0.018, 0.037, and 0.026, respectively (Fig. 3b).

### Correlations between 16S rRNA gene sequencing and LC-MS untargeted metabolomics.

**FIG 5:**
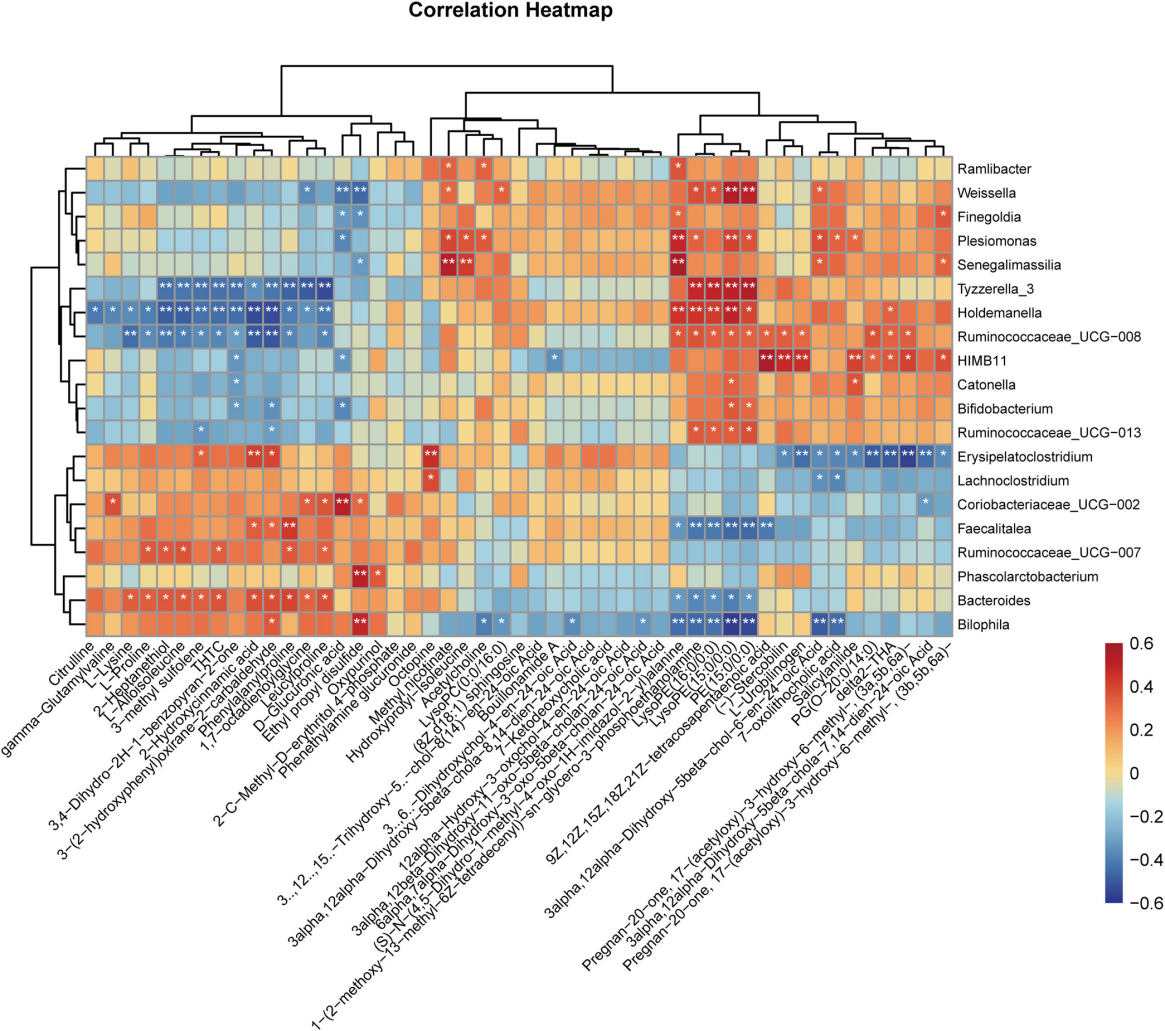
Heat map visualization of the correlation analysis for the top 50 differential metabolites and the differential bacteria between day 0 and day 180 of the voyage. Spearman correlation analysis was used to examine correlations between the differential bacteria and the top 50 differential fecal metabolites according to their relative abundances.

**FIG 6:**
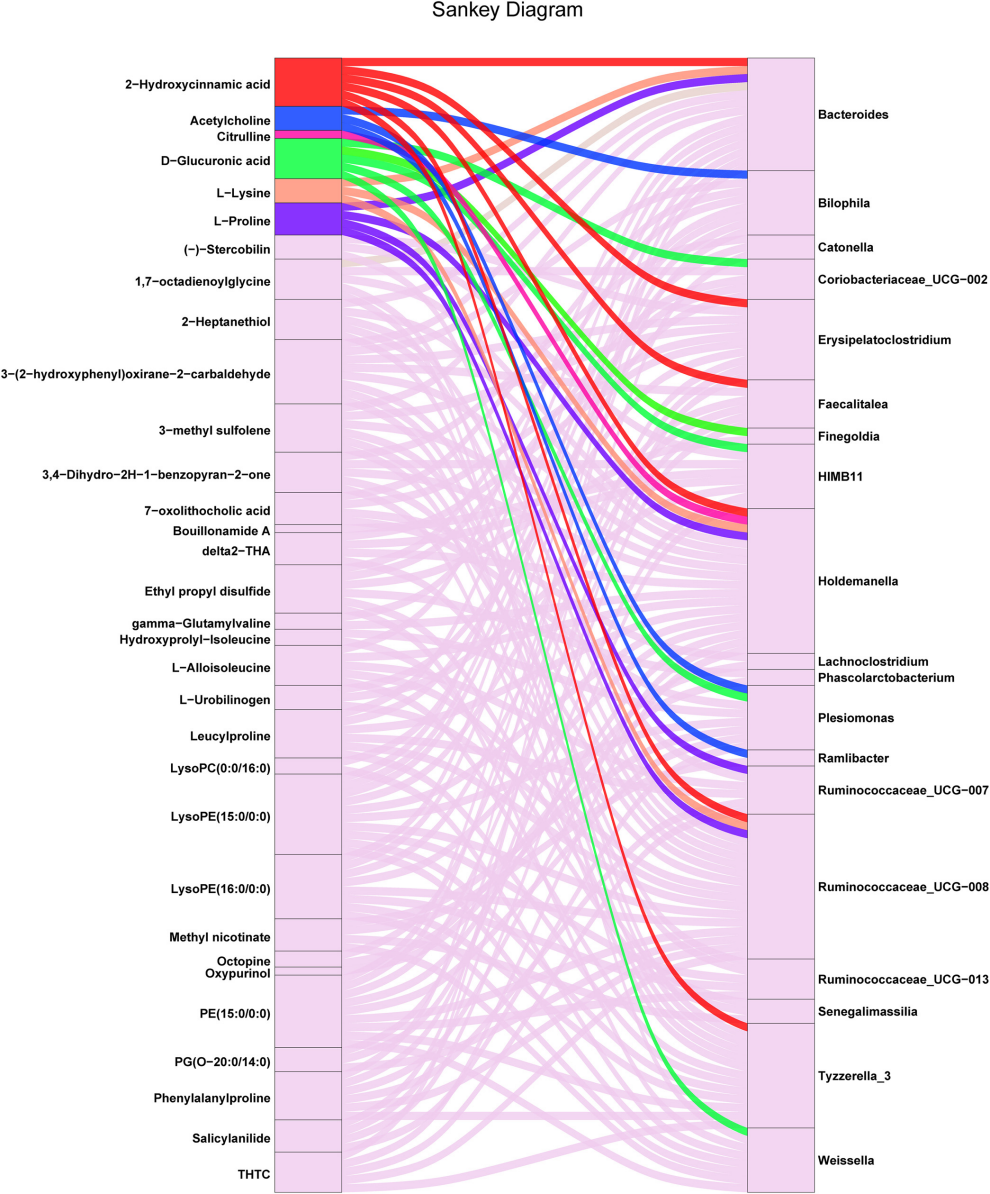
Sankey diagram showing the correlations between the differential metabolites and their correlated gut microbes. The Sankey diagram was constructed based on the correlation results for the differential microbiota members-top 50 differential metabolites; a *P* value of <0.05 was chosen to select correlations. The six differential metabolites (in colors) annotated in human KEGG pathways were correlated with the 14 differential microbiota members. The differential metabolites in the background color were not identified in human KEGG pathways. The total rectangle size of each bacterium/metabolite represents the number of correlations between each metabolite and its correlated microbiota.

To investigate the associations among the gut microbiome, fecal metabolites, and human metabolic pathways, Spearman correlation analysis was first employed to evaluate the links between the differential bacteria and the top 50 differential fecal metabolites according to their relative abundances (Fig. 5). Out of the top 50 differential fecal metabolites, 6 could be annotated according to the human KEGG pathway mapper. The correlations among the differential fecal metabolites, altered bacteria, and related human metabolic pathways were then tabulated (Table 2). Close relationships were observed between 14 differential microbiota members and six differential fecal metabolites that might affect specific human metabolic pathways. Specifically, the increase in fecal acetylcholine was correlated with *Bilophila*, *Plesiomonas*, and *Ramlibacter*, which might impact the cholinergic synapse. Amino acids such as l-proline and l-lysine, which were significantly decreased, were correlated with *Ruminococcaceae*_UCG-008, *Holdemanella*, and *Bacteroides*, which might influence protein digestion and absorption as well as aminoacyl-tRNA biosynthesis. Furthermore, the decrease in citrulline was correlated with *Holdemanella*, which might influence arginine biosynthesis. The decrease in d-glucuronic acid was correlated with *Weissella*, *Plesiomonas*, HIMB11, *Finegoldia*, and *Coriobacteriaceae*_UCG-002, which might influence pentose and glucuronate interconversions. The decrease in 2-hydroxycinnamic acid was correlated with *Ruminococcaceae*_UCG-008, *Holdemanella*, *Tyzzerella*_3, *Bacteroides*, *Faecalitalea*, and *Erysipelatoclostridium*, which might influence phenylalanine metabolism. Furthermore, a Sankey diagram was drawn to visualize the covariation between the differential metabolites and their correlated gut microbes (Fig. 6). *Holdemanella* was found to be associated with the greatest number of decreased differential metabolites, followed by *Bacteroides* and *Ruminococcaceae*_UCG-008. Specifically, *Holdemanella* was correlated with citrulline, l-lysine, l-proline, and 2-hydroxycinnamic acid, which might impact human protein digestion and absorption as well as phenylalanine metabolism. In other words, these species could perform pivotal and pleiotropic roles in the microbiome-host interaction.

**TABLE 2:** Correlations among differential bacteria, differential fecal metabolites, and annotated human metabolic pathways

Differential metabolite	Metabolite change	Differential microbiota member	Microbiota member change	Rho	Correlation *P* value	Human pathway annotation(s)
Acetylcholine	↑	*Bilophila*	↓	−0.396	0.013	Cholinergic synapse
		*Plesiomonas*	↑	0.353	0.027	
		*Ramlibacter*	↑	0.329	0.041	

d-Glucuronic acid	↓	*Weissella*	↑	−0.418	0.008	Pentose and glucuronate interconversions
		*Plesiomonas*	↑	−0.372	0.019	
		HIMB11	↑	−0.340	0.034	
		*Finegoldia*	↑	−0.327	0.042	
		*Coriobacteriaceae*_UCG-002	↓	0.509	0.0009	

Citrulline	↓	*Holdemanella*	↑	−0.407	0.010	Arginine biosynthesis

l-Lysine	↓	*Ruminococcaceae*_UCG-008	↑	−0.449	0.004	Protein digestion and absorption, aminoacyl-tRNA biosynthesis, lysine degradation
		*Holdemanella*	↑	−0.364	0.023	
		*Bacteroides*	↓	0.334	0.038	

l-Proline	↓	*Ruminococcaceae*_UCG-008	↑	−0.359	0.025	Protein digestion and absorption, aminoacyl-tRNA biosynthesis
		*Holdemanella*	↑	−0.396	0.013	
		*Bacteroides*	↓	0.339	0.035	
		*Ruminococcaceae*_UCG-007	↓	0.352	0.028	

2-Hydroxycinnamic acid	↓	*Ruminococcaceae*_UCG-008	↑	−0.494	0.001	Phenylalanine metabolism
		*Holdemanella*	↑	−0.534	0.0005	
		*Tyzzerella*_3	↑	−0.354	0.027	
		*Bacteroides*	↓	0.372	0.020	
		*Faecalitalea*	↓	0.345	0.031	
		*Erysipelatoclostridium*	↓	0.412	0.009	

**FIG 7:**
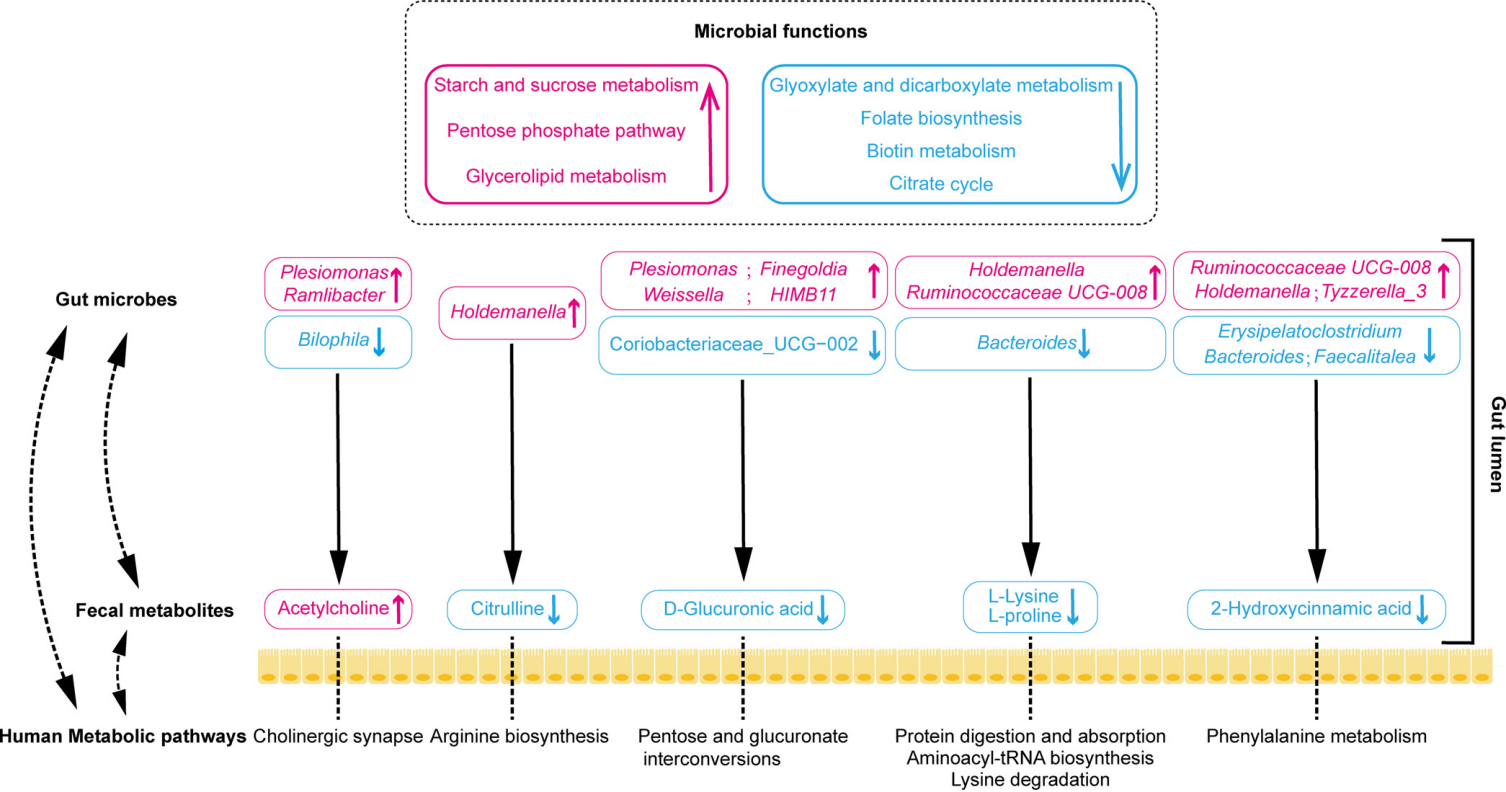
Schematic overview of gut microbiota-host interactions during the 6-month voyage. Pink text indicates increased potential pathobionts, fecal metabolites, and microbial functions, while blue text indicates decreased potential probiotics, fecal metabolites, and microbial functions.

Taken together, these findings indicate that long-term voyages could induce gut dysbiosis and might influence human metabolic pathways. A schematic overview was constructed to depict the gut microbiome-host interactions due to the long-term voyage (Fig. 7).

## DISCUSSION

In this study, we performed a multi-omics analysis of 16S rRNA gene sequencing data and LC-MS untargeted metabolomics to analyze the impacts of a 6-month voyage on seafarers. The major findings of this study are as follows: (i) the long-term voyage significantly altered the composition of the gut microbiota; (ii) 20 differential metabolites within six differentially enriched human metabolic pathways are worthy of attention; and (iii) Spearman correlation analysis showed close relationships between 14 differential microbiota members and six differential fecal metabolites, which might affect specific human metabolic pathways.

During a long-term voyage, seafarers are confronted with various physical and psychological stressors. Consistent with previous reports (17, 18), the current study revealed that the gut microbial composition of seafarers was significantly altered after a long-term voyage. Predicted microbial functions, including folate biosynthesis and biotin metabolism, were significantly decreased after the voyage. Both folate and biotin are classified as B vitamins; they are not only absorbed from the diet via the small intestine but also biosynthesized by the gut microbiota and absorbed in the distal gut. B vitamins function as nutrient benefactors and immunoregulators in the body and modulate the gut microbiota (20). Folate, also known as vitamin B_9_, can be biosynthesized by *Bacteroides*, *Bifidobacterium*, Streptococcus, and *Lactococcus* spp. Folate is a carrier and donor of one-carbon units, participating in DNA synthesis and maintenance (21). Biotin, also known as vitamin B_7_, can be biosynthesized by *Bacteroides* and is involved in cellular metabolic pathways; it also has immunological and inflammatory functions (22). An insufficient supply of fresh fruits and vegetables during long-term voyages may result in decreases in folate biosynthesis and biotin metabolism due to a lack of B vitamins, which can increase the risk of B vitamin deficiencies and related illnesses such as megaloblastic anemia and neurological and neuropsychiatric disorders. A recent study showed that 120-day ocean sailing decreased the serum folic acid concentrations and the mean corpuscular hemoglobin levels in seafarers (19).

The LC-MS untargeted metabolomics analysis identified 20 differential metabolites within six differentially enriched metabolic pathways (arginine biosynthesis, lysine degradation, phenylalanine metabolism, sphingolipid metabolism, pentose and glucuronate interconversions, and glycine, serine, and threonine metabolism) in the seafarers. In order to obtain more detailed information on the microbiome-host interaction, a Spearman correlation analysis of the differential microbiota members and differential metabolites was performed. The results revealed that 14 differential microbiota members were closely correlated with six differential fecal metabolites, which might have specific effects on the metabolic pathways of seafarers. Specifically, decreases in various fecal metabolites (including d-glucuronic acid, citrulline, l-lysine, and l-proline) were correlated with various differential bacteria (including *Holdemanella*, *Ruminococcaceae*_UCG-008, and *Bacteroides*), which might impact the human metabolic pathways related to pentose and glucuronate interconversions, arginine biosynthesis, and lysine and proline metabolism.

Two differential bacteria, *Plesiomonas* and *Holdemanella*, identified in our study might have insidious effects on seafarers during long sea voyages. *Plesiomonas*, a food- and waterborne pathogen, can cause bacterial gastroenteritis characterized by diarrhea, bacteremia, and other ailments (23). *Plesiomonas* infection is strongly associated with the consumption of uncooked shellfish, raw oysters, and shrimp. *Plesiomonas* is considered a traveler’s diarrhea pathogen and is frequently observed in travelers to high-risk regions, including South Asia, Southeast Asia, Latin America, and Africa (24). Therefore, infection with *Plesiomonas* during a long-term voyage poses significant risks. The results of the current study indicated that an increased abundance of *Plesiomonas* was positively correlated with acetylcholine; *Plesiomonas* might act on the cholinergic synapse, and this may be one of its potential pathogenic mechanisms. Changes to neurotransmitters derived from the gut might have adverse effects on gastrointestinal functions and neuropsychiatric health. First, the central nervous system (including the sensory neurons of the dorsal root ganglion and vagus nerve and neurons in the autonomic nervous system) and the enteric nervous system (including the submucosal plexus and myenteric plexus) can receive signals from nicotinic acetylcholine receptors, perceive the gut status, regulate alimentary tract functions, and modulate the immune response (25). In response to intraluminal bacterial stimuli, afferent neurons are activated, and submucosal secretomotor neurons are then stimulated to secrete vasoactive intestinal peptides and acetylcholine (which binds to muscarinic receptors on epithelial cells). Moreover, the activation of enteric interneurons or extrinsic efferent fibers can also release acetylcholine and stimulate nicotinic receptors present on secretomotor neurons. Enterocyte secretion is considered to be the predominant mechanism responsible for acute infectious diarrhea (26). However, it is worth noting that foods containing considerable amounts of acetylcholine (e.g., aubergine, spinach, peas, and mung beans) might also have physiological or pathophysiological effects on the human body (27). Further studies are needed to demonstrate the relationship between the gut microbiota identified in our study and fecal acetylcholine.

Furthermore, *Holdemanella* may influence brain function and behavior via the microbiota-gut-brain axis. Chen et al. reported previously that *Holdemanella* was related to a risk of anxiety (β = −0.008; *P* = 4.20 × 10^−3^) and depression (β = −0.007; *P* = 1.39 × 10^−2^) (28). Scheepers et al. investigated the associations between natural compulsive-like behavior in deer mice and alterations in the gut microbiota and found that the prevalence of *Holdemanella* was associated with obsessive-compulsive disorder, a disease driven by aberrant cognitive processes (29). The current study revealed a strong negative correlation between *Holdemanella* and 2-hydroxycinnamic acid, and this might influence phenylalanine metabolism. The inadequate conversion of phenylalanine can lead to hyperphenylalaninemia, a disorder that can impair cerebral function and cause neuropsychiatric disorders (30, 31). Furthermore, *Holdemanella* was negatively correlated with citrulline, l-lysine, and l-proline, which might influence human arginine and proline metabolism.

Additionally, *Bacteroides* may be a potential probiotic for seafarers. *Bacteroides* is involved in the production and utilization of short-chain fatty acids (SCFAs) (32). SCFAs are important energy sources for intestinal epithelial cells, and they also act as inhibitors of histone deacetylases and ligands for G-protein-coupled receptors. Thus, they can exert anti-inflammatory effects by regulating NF-κB activity, peripheral T cell function, and proinflammatory innate immune responses (33–35). Hsiao et al. reported previously that Bacteroides fragilis can correct gut permeability and ameliorate autism spectrum disorder pertaining to communicative, stereotypic, anxiety-like, and sensorimotor behaviors in mice (36). Our study showed that the significant decrease in *Bacteroides* not only influenced the *Firmicutes*/*Bacteroidetes* ratio but also was correlated with the abundance of metabolites (e.g., l-proline, l-lysine, and 2-hydroxycinnamic acid) in feces, which might impact human metabolic pathways, including protein digestion and absorption, aminoacyl-tRNA biosynthesis, lysine degradation, and phenylalanine metabolism.

The current study is the first to reveal the intestinal microbiome-host interactions after a long-term sea voyage by combining gut microbiome and metabolomics analyses. Preventative measures, such as adjusting the gut microbiota by decreasing potential pathobionts (e.g., *Holdemanella* and *Plesiomonas*) or increasing potential probiotics (e.g., *Bacteroides*) as well as offsetting the decrease in B vitamins (e.g., folate and biotin) and metabolites (e.g., d-glucuronic acid, citrulline, l-lysine, and l-proline) via dietary adjustment or nutritional supplements, might be beneficial to the health of seafarers during long-term sea voyages. However, the limitations of this study should be noted. First, other critical microorganisms aside from bacteria, such as viruses, bacteriophages, yeasts, and fungi, also influence microorganism-host interactions and could be implicated in the occurrence of disorders and diseases. Second, although hypotheses were made about several potentially affected human metabolic pathways, direct causality among the differential microbes, differential metabolites, and altered human metabolic pathways could not be established. Whether and how the microbiota influences the host during a long-term voyage remain to be further investigated in future research. Third, the environment faced by seafarers during long-term navigation is complex, and it is quite difficult to control for factors such as individual habits (e.g., diet, sleep, and activity), endogenous stressors (e.g., insomnia and the circadian rhythm), and exogenous stressors (e.g., different climate zones and chemical exposures). Fourth, due to the limited medical facilities on board, blood biochemistry could not be fully determined, and diagnostic imaging could not be performed; thus, disease diagnosis was limited. The primary focus of this study was on the overall impacts of a long-term voyage on the human gut microbiome and metabolome. This study provides valuable clues regarding the physiological and psychological disorders that occur during long-term voyages, which could be reflected in changes to the gut microbiome and metabolome. In the future, prospective studies of long-term voyages should analyze multiple psychological and physiological scales. Furthermore, cellular and animal studies are required to verify the underlying causality among long-term voyages, physical and psychological disorders, and the gut microbiome/metabolome. In conclusion, 16S rRNA gene sequencing and LC-MS untargeted metabolomics were employed in this study to clarify the influence of a 6-month voyage on seafarers and to unveil the microbiome-host interactions. These findings indicated that the gut microbiome was significantly altered after the voyage, and changes in several human metabolic pathways were implicated. However, it is critical to note that this study is a preliminary pilot project with a limited sample size. Thus, these results should be verified in future studies.

## MATERIALS AND METHODS

### Voyage and participant information.

All relevant aspects of the study were approved by the ethics committee of the Navy Military Medical University, Shanghai, China. Informed consent was obtained from each subject, and the study protocol was performed in accordance with the ethical guidelines of the 1975 Declaration of Helsinki. In total, 30 participants were enrolled in this study. All participants were male, aged 18 to 35 years, and were participating in a sea voyage lasting 6 months. The voyage occurred from 12 June to 27 December 2017. During the voyage, there was a uniform supply of food and drink. The standard diet was $30 per day and included frozen meat, vegetables, fruits, and canned food, etc. The ship docked on average once every 2 weeks to replenish the supply of fresh food and drinks. Due to storage problems, the supply of fresh vegetables and fruit was limited. The seafarers were housed in small closed rooms on the ship; each room was approximately 10 m^2^ and housed six seafarers. Fresh fecal samples were collected from all participants for 16S rRNA gene sequencing and LC-MS untargeted metabolomic analysis. All participants were healthy.

### Fecal sample collection.

Fresh fecal samples were collected using sterile sampling tubes before and after the voyage. The samples were stored at −80°C in a freezer for 16S rRNA gene sequencing and LC-MS untargeted metabolomics analysis.

### 16S rRNA gene sequencing.

Samples were snap-frozen and stored at −80°C after collection. Bacterial DNA was isolated using a DNeasy PowerSoil kit (Qiagen, Hilden, Germany) according to the manufacturer’s instructions. The DNA concentration and integrity were measured by a NanoDrop 2000 spectrophotometer (Thermo Fisher Scientific, Waltham, MA, USA) and agarose gel electrophoresis, respectively. PCR amplification of the V3-V4 hypervariable regions of the bacterial 16S rRNA gene was carried out in a 25-μL reaction mixture using universal primer pairs (343F [5′-TACGGRAGGCAGCAG-3′] and 798R [5′-AGGGTATCTAATCCT-3′]). The reverse primer contained a sample barcode, and both primers were connected with an Illumina sequencing adapter. The amplicon quality was visualized using gel electrophoresis. The PCR products were purified with Agencourt AMPure XP beads (Beckman Coulter Co., USA) and quantified using a Qubit double-stranded DNA (dsDNA) assay kit. The concentrations were then adjusted for sequencing. Sequencing was performed on an Illumina MiSeq platform with two paired-end read cycles of 300 bases each (Illumina Inc., San Diego, CA, and OE Biotech Company, Shanghai, China).

### Bioinformatic analysis of 16S rRNA gene sequencing.

Trimmomatic software (version 0.35) was used to cut off ambiguous bases (N’s) and low-quality sequences with an average quality score of <20 using the sliding-window trimming approach (37). Next, FLASH software (version 1.2.11) was used to assemble paired-end reads (38). QIIME software (version 1.8.0) was used to denoise sequences and remove reads with chimera (39). The primer sequences of the clean reads were removed and then clustered to generate OTUs using Vsearch software (version 1.4.2) with a 97% similarity cutoff (40). The QIIME package was used to select representative reads for each OTU. Next, these representative reads were annotated, and a BLAST search was performed against the Silva database (version 123) by the RDP classifier with a confidence threshold of 70% (41). A phylogenetic tree of representative sequences was constructed using pynast (version 0.1) (42). The Chao1, Shannon, Simpson, and coverage indices were calculated to assess alpha diversity (Wilcoxon rank sum test). NMDS analysis based on the weighted UniFrac distance algorithm and the ANOSIM method was used to assess beta diversity. Multivariate statistics were performed. ANOSIM was used to test the differences between the start (day 0) and end (day 180) of the voyage, while the Wilcoxon algorithm and linear discriminant analysis (LDA) coupled with effect size measurements (LEfSe) were used to analyze the species differences between the start (day 0) and end (day 180) of the voyage. PICRUSt2 functional prediction was performed using PICRUSt2 software (43). Based on the results of PICRUSt2, Welch’s *t* test was performed using STAMP software to analyze the differential pathways. Pathways with *P* values of <0.05 were chosen, and the top 20 differential pathways were plotted for the predicted microbial functional analysis.

### Metabolite detection and bioinformatic analysis of LC-MS metabolomics.

All chemicals and solvents were of analytical or high-performance liquid chromatography (HPLC) grade. Water, methanol, acetonitrile, and formic acid were purchased from CNW Technologies GmbH (Düsseldorf, Germany). l-2-Chlorophenylalanine was purchased from Shanghai Hengchuang Bio-technology Co., Ltd., (Shanghai, China). Sixty milligrams of an accurately weighed sample was transferred to a 1.5-mL Eppendorf tube. QC samples were prepared by mixing aliquots of all samples to obtain a pooled sample. Next, 20 μL of an internal standard (2-chloro-l-phenylalanine in methanol [0.3 mg/mL]) was added to each sample. The metabolites from each sample were collected, filtered through 0.22-μm microfilters, and transferred to LC vials, which were stored at −80°C until LC-MS analysis.

A DionexUltimate 3000 RS ultrahigh-performance liquid chromatography (UHPLC) system fitted with a Q Exactive Quadrupole-Orbitrap mass spectrometer equipped with a heated electrospray ionization (ESI) source (Thermo Fisher Scientific, Waltham, MA, USA) was used for metabolic profiling; profiling was performed in both ESI-positive and ESI-negative ion modes. An Acquity ultraperformance liquid chromatography (UPLC) ethylene-bridged hybrid (BEH) C_18_ column (1.7 μm, 2.1 by 100 mm) was employed in both positive and negative modes. The binary gradient elution system used for chromatographic separation, consisted of water (containing 0.1% [vol/vol] formic acid) (solvent A) and acetonitrile (containing 0.1% [vol/vol] formic acid) (solvent B), and separation was achieved using the following gradient: 5 to 20% B over 0 to 2 min, 20 to 60% B over 2 to 4 min, 60 to 100% B over 4 to 11 min, a hold at 100% B for 2 min, 100% to 5% B over 13 to 13.5 min, and a hold at 5% B over 13.5 to 14.5 min. The flow rate was 0.4 mL/min, and the column temperature was 45°C. All samples were kept at 4°C during analysis. The injection volume was 5 μL. The mass range was from *m/z* 66.7 to 1,000.5. The resolutions were set at 70,000 for the full MS scans and 35,000 for high-energy collisional dissociation (HCD) tandem MS (MS/MS) scans. The collision energies were set at 10, 20, and 40 eV. The mass spectrometer was operated as follows: spray voltages of (3000V in positive ion mode and 2500V in negative ion mode), a sheath gas flow rate of 45 arbitrary units, an auxiliary gas flow rate of 15 arbitrary units, and a capillary temperature of 350°C. The QCs were injected at regular intervals (every 10 samples) throughout the analytical run to provide a set of data from which repeatability could be assessed.

Metabolites were identified by progenesis QI data processing software (Waters Corporation, Milford, MA, USA), based on public databases such as the Human Metabolome Database (http://www.hmdb.ca/) and Lipid Maps (http://www.lipidmaps.org/) and self-built databases. The positive and negative data were combined and imported into the R ropls package. OPLS-DA was performed to visualize the metabolic changes between the start (day 0) and end (day 180) of the voyage after mean centering (Ctr) and Pareto variance (Par) scaling. A variable importance in projection (VIP) score of >1 was considered relevant for discrimination between the start (day 0) and end (day 180) of the voyage. The identification of differential metabolites was based on a VIP score of >1.0 from the OPLS-DA model and a *P* value of <0.05 from two-tailed Student’s *t* test on the normalized peak areas. Based on the hypergeometric distribution, KEGG pathway enrichment analysis of differential electrochemical mass spectrometry (DEM) was performed to screen the significantly enriched terms (*P < *0.05) using the R clusterProfiler package. The R clusterProfiler package was used to draw a bubble plot of the top 20 enrichment terms:

$$P=1-\overset{m-1}{\underset{i=0}{\sum }}\frac{\left(\begin{array}{c} M\\ i \end{array}\right)\left(\begin{array}{c} N-M\\ n-i \end{array}\right)}{\left(\begin{array}{c} N\\ n \end{array}\right)}$$

where *N* represents the total number of metabolites, *n* represents the number of differential metabolites among *N*, *M* represents the number of metabolites annotated for a particular pathway, and *m* represents the number of differential metabolites for a particular pathway. A *P* value of <0.05 was used as the threshold.

### Statistical analysis.

The data were analyzed using SPSS statistical software (version 21; IBM Corp., NY, USA) and R (version 3.2.0; R Foundation for Statistical Computing, Vienna, Austria). Normally distributed quantitative data are presented as means ± standard deviations (SD), and nonnormally distributed data are presented as interquartile ranges (25%, 75%). Wilcoxon rank sum tests were used to analyze the differences in the microbiota, and two-tailed Student’s *t* tests were used to analyze the differences in metabolites between the start (day 0) and end (day 180) of the voyage. The Benjamini-Hochberg false discovery rate (FDR) method (44) was applied to the differential abundance analysis of the microbiota and metabolites for multiple-hypothesis testing correction. Spearman correlation analysis was used to analyze the correlations between differential bacteria and the top 50 differential fecal metabolites according to their relative abundances. A *P* value of <0.05 was considered statistically significant.

### Data availability.

The microbiome raw data have been deposited in the NCBI database under accession number PRJNA860006, and metabolomics raw data have been deposited in the MetaboLights database (www.ebi.ac.uk/metabolights/studies) under study identifier MTBLS5335. All data that support the findings of this study are included in the manuscript and the supplemental material.
