# Supplementary material for: Alterations in the Gut Microbiota and Metabolomics of Seafarers after a Six-Month Sea Voyage
Source: Microbiol Spectr. 2022 Oct 5;10(5):e01899-22. doi: 10.1128/spectrum.01899-22 (PMC9603232; doi:10.1128/spectrum.01899-22)
Supplement: Supplemental file 1 — Supplemental material. Download spectrum.01899-22-s0001.pdf, PDF file, 0.2 MB [file spectrum.01899-22-s0001.pdf]

Supplement Figure

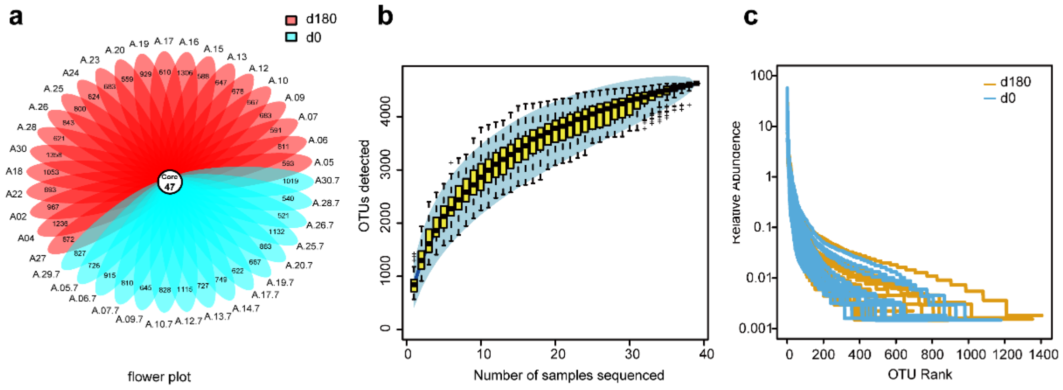

Figure S1. 16S rRNA gene sequencing of bacteria in feces. (a) OTU flower plot between d0 and d180 of the voyage. The center of the flower plot is the core OTUs which represent the common OTUs between d0 and d180 of the voyage. The figure in the petal means the total OTUs of each sample minus the common OTUs in the center. **(b)** Specaccum curve of all sequenced samples. The horizontal axis represents the sample size, and the vertical axis represents the number of OTUs detected. **(c)** Rank-Abundance curve between d0 and d180 of the voyage. On the horizontal axis, OTUs are sorted according to the number of sequences they contain. For example, "500" represents the 500th abundance OTU in the sample; The vertical axis represents the relative abundance of the OTU, for example, "0.01" represents 0.01 %.

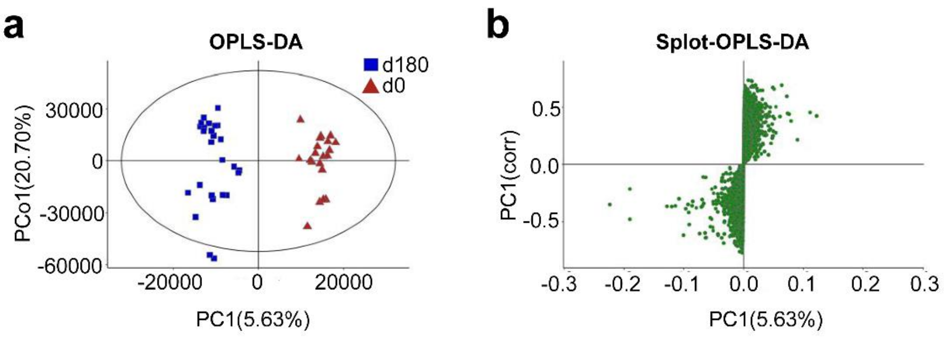

Figure S2. Liquid chromatography-mass spectrometry analysis of fecal metabolites. (a) Orthogonal partial least squares discrimination analysis (OPLS-DA) between d0 and d180 of the voyage. **(b)** OPLS-DA S-plot. The horizontal axis represents the characteristic values of the effects of metabolites between d0 and d180 of the voyage, and the vertical axis represents the correlations between the sample scores and the metabolites. The closer the metabolites were to the upper right corner and the lower left corner, the greater the difference was.
